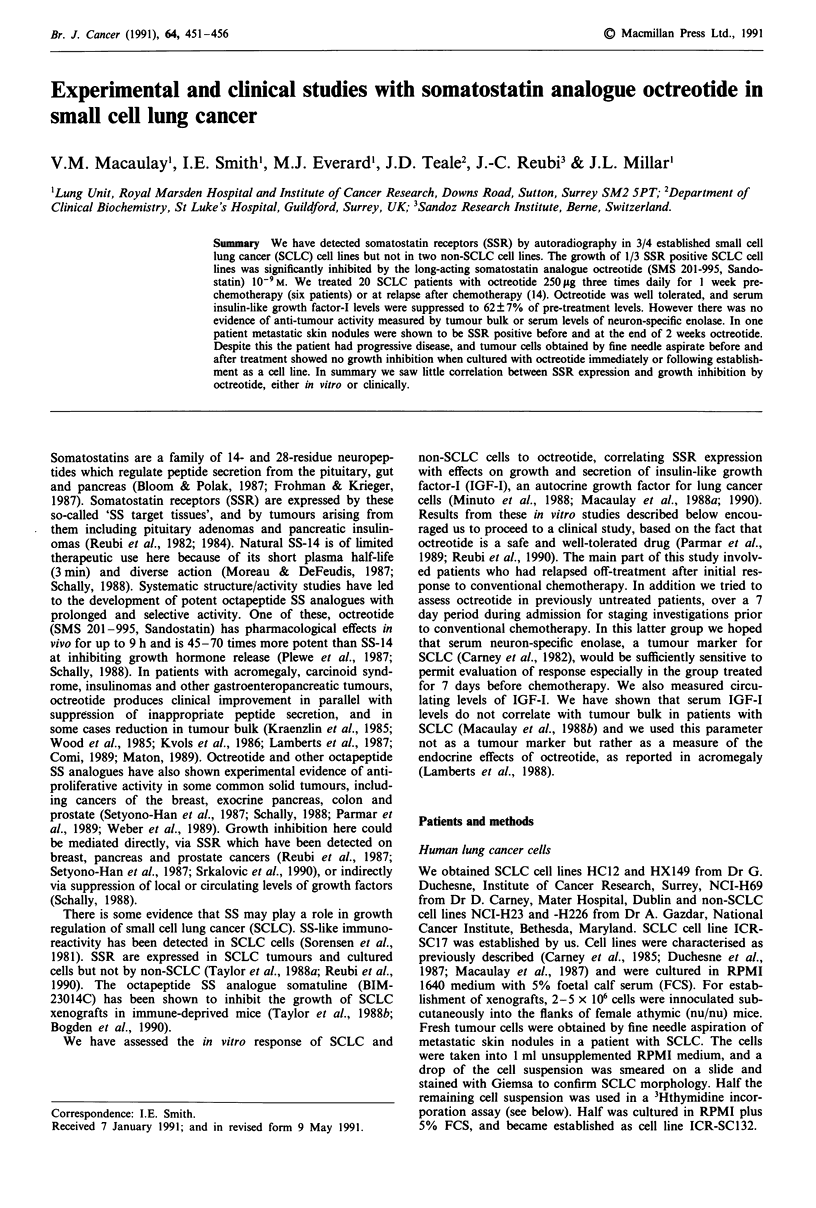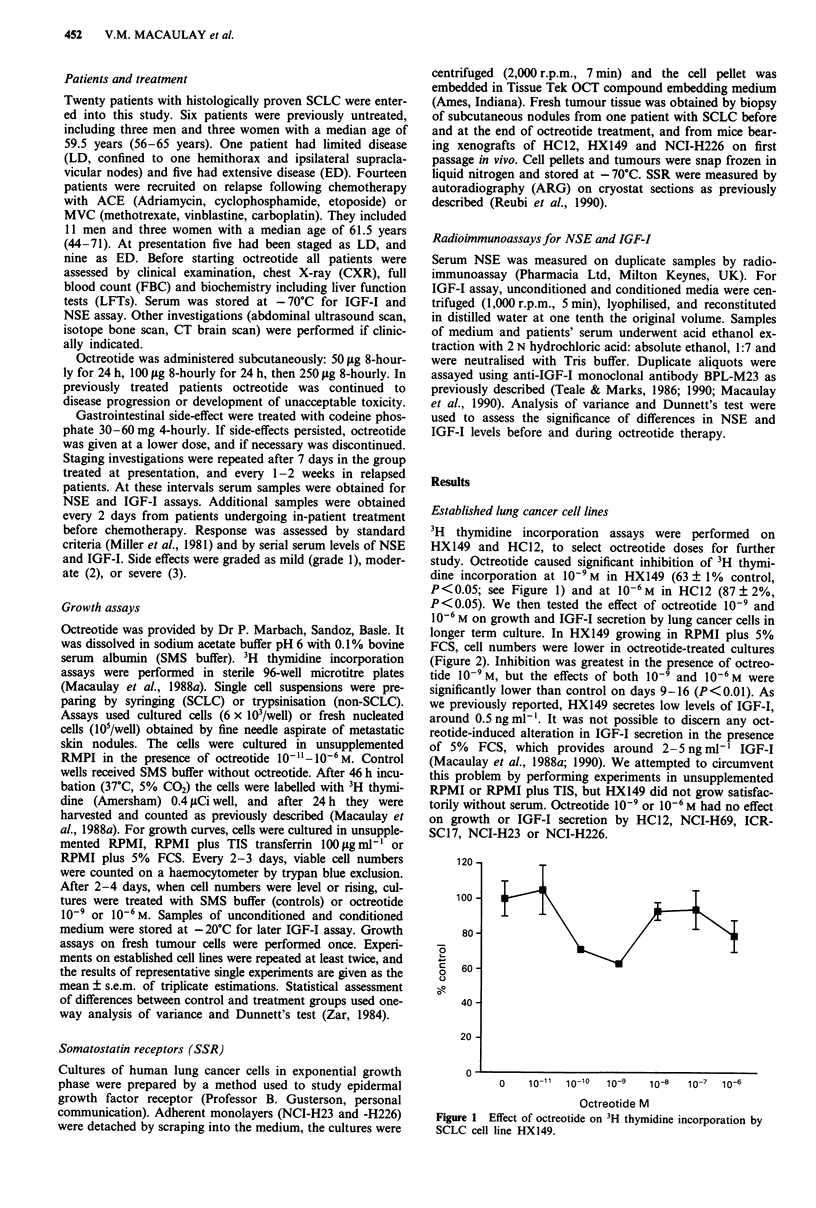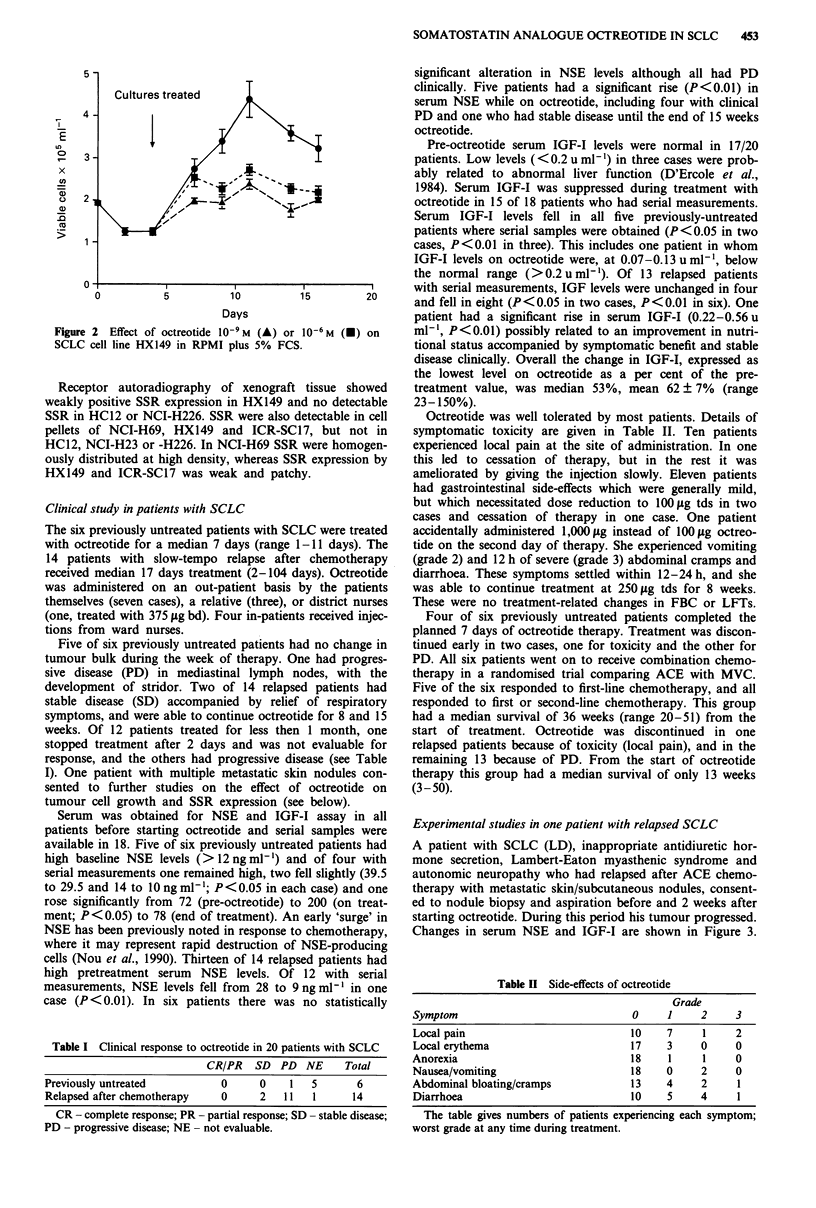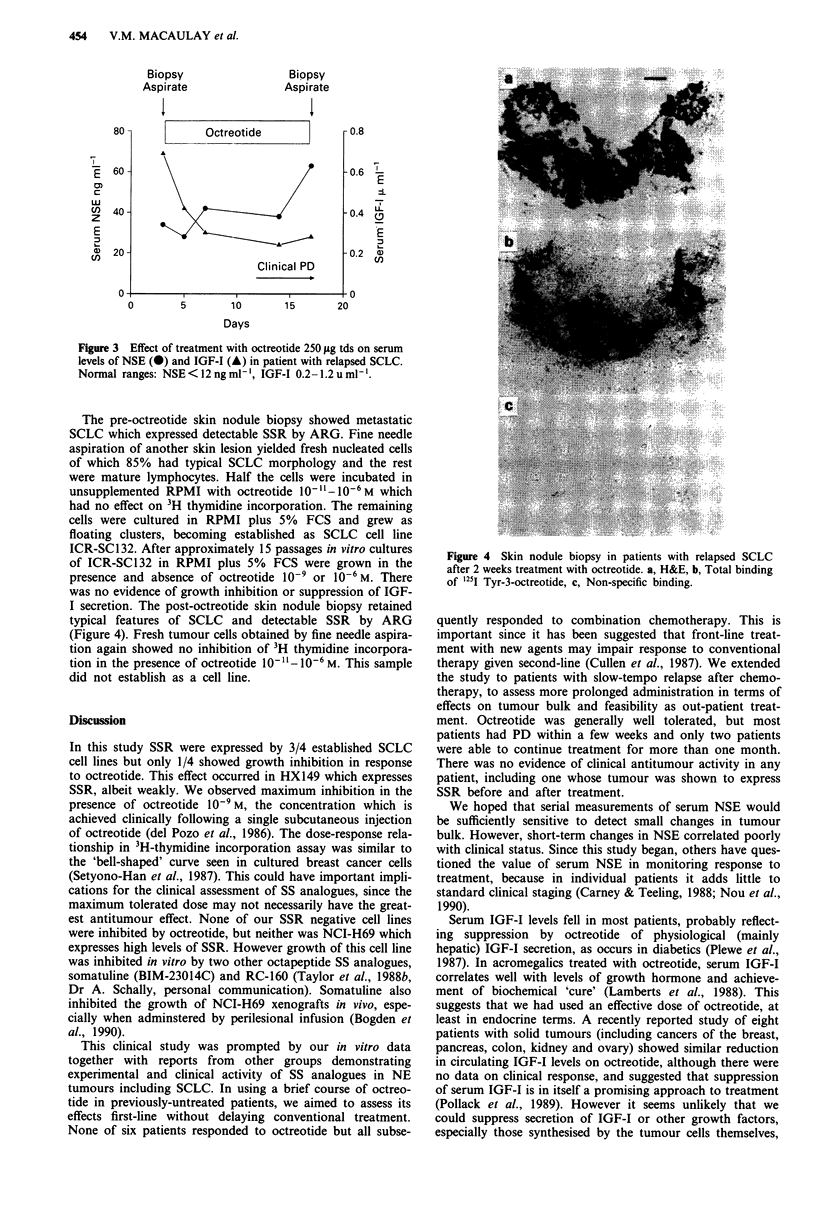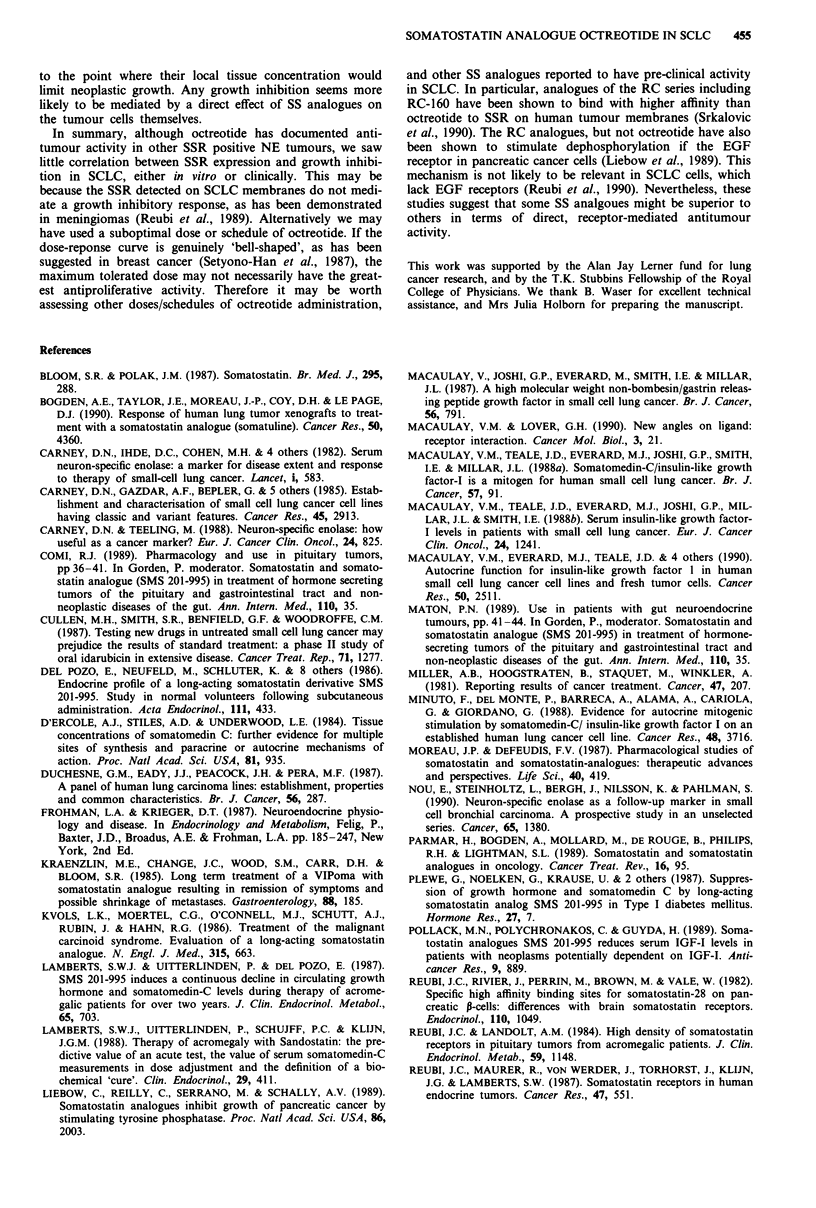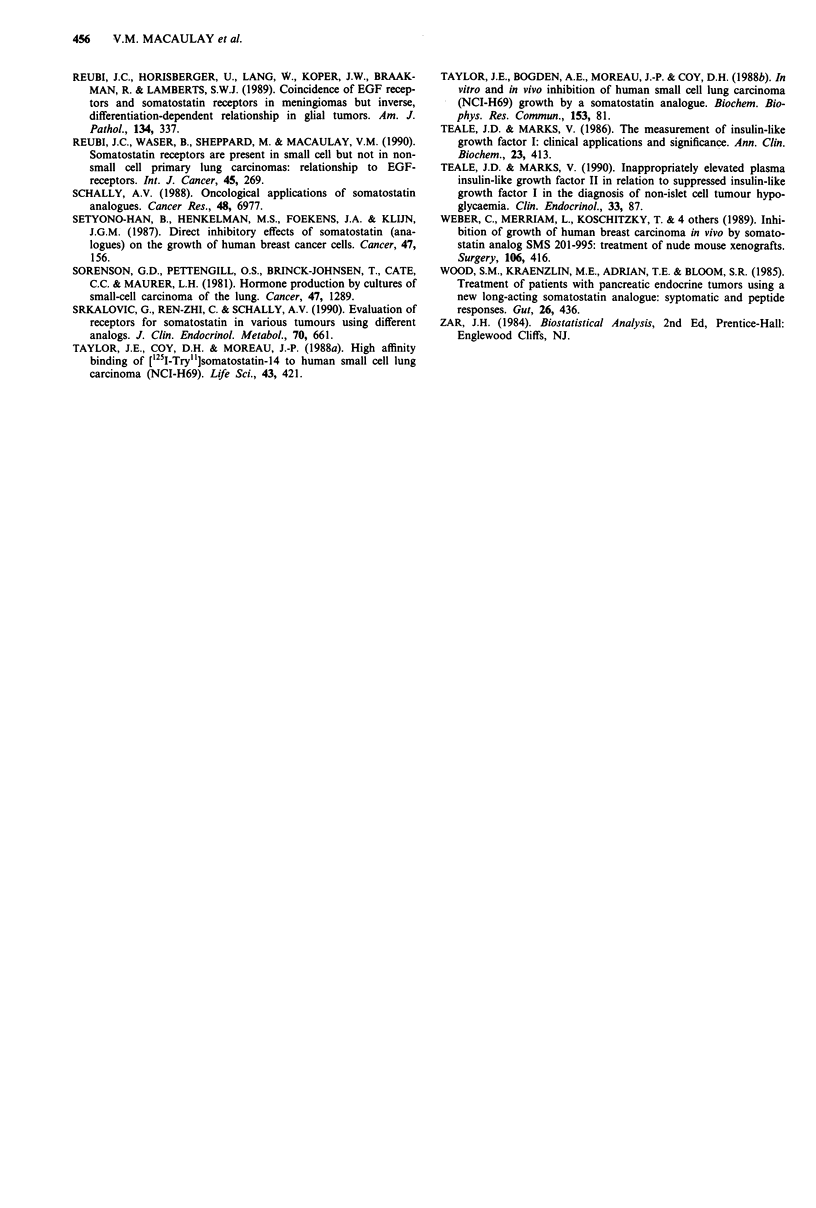# Experimental and clinical studies with somatostatin analogue octreotide in small cell lung cancer.

**DOI:** 10.1038/bjc.1991.330

**Published:** 1991-09

**Authors:** V. M. Macaulay, I. E. Smith, M. J. Everard, J. D. Teale, J. C. Reubi, J. L. Millar

**Affiliations:** Lung Unit, Royal Marsden Hospital, Sutton, Surrey, UK.

## Abstract

**Images:**


*Br. J. Cancer* (1991), **64**, 451–456 © Macmillan Press Ltd., 1991

### Experimental and clinical studies with somatostatin analogue octreotide in small cell lung cancer

V.M. Macaulay[1], I.E. Smith[1], M.J. Everard[1], J.D. Teale[2], J.-C. Reubi[3] & J.L. Millar[1]

[1]*Lung Unit, Royal Marsden Hospital and Institute of Cancer Research, Downs Road, Sutton, Surrey SM2 5PT;* [2]*Department of Clinical Biochemistry, St Luke's Hospital, Guildford, Surrey, UK;* [3]*Sandoz Research Institute, Berne, Switzerland.*

**Summary** We have detected somatostatin receptors (SSR) by autoradiography in 3/4 established small cell lung cancer (SCLC) cell lines but not in two non-SCLC cell lines. The growth of 1/3 SSR positive SCLC cell lines was significantly inhibited by the long-acting somatostatin analogue octreotide (SMS 201-995, Sandostatin) $10^{-9}$ M. We treated 20 SCLC patients with octreotide 250 µg three times daily for 1 week pre-chemotherapy (six patients) or at relapse after chemotherapy (14). Octreotide was well tolerated, and serum insulin-like growth factor-I levels were suppressed to 62±7% of pre-treatment levels. However there was no evidence of anti-tumour activity measured by tumour bulk or serum levels of neuron-specific enolase. In one patient metastatic skin nodules were shown to be SSR positive before and at the end of 2 weeks octreotide. Despite this the patient had progressive disease, and tumour cells obtained by fine needle aspirate before and after treatment showed no growth inhibition when cultured with octreotide immediately or following establishment as a cell line. In summary we saw little correlation between SSR expression and growth inhibition by octreotide, either *in vitro* or clinically.

Somatostatins are a family of 14- and 28-residue neuropeptides which regulate peptide secretion from the pituitary, gut and pancreas (Bloom & Polak, 1987; Frohman & Krieger, 1987). Somatostatin receptors (SSR) are expressed by these so-called 'SS target tissues', and by tumours arising from them including pituitary adenomas and pancreatic insulinomas (Reubi *et al.*, 1982; 1984). Natural SS-14 is of limited therapeutic use here because of its short plasma half-life (3 min) and diverse action (Moreau & DeFeudis, 1987; Schally, 1988). Systematic structure/activity studies have led to the development of potent octapeptide SS analogues with prolonged and selective activity. One of these, octreotide (SMS 201–995, Sandostatin) has pharmacological effects *in vivo* for up to 9 h and is 45–70 times more potent than SS-14 at inhibiting growth hormone release (Plewe *et al.*, 1987; Schally, 1988). In patients with acromegaly, carcinoid syndrome, insulinomas and other gastroenteropancreatic tumours, octreotide produces clinical improvement in parallel with suppression of inappropriate peptide secretion, and in some cases reduction in tumour bulk (Kraenzlin *et al.*, 1985; Wood *et al.*, 1985; Kvols *et al.*, 1986; Lamberts *et al.*, 1987; Comi, 1989; Maton, 1989). Octreotide and other octapeptide SS analogues have also shown experimental evidence of anti-proliferative activity in some common solid tumours, including cancers of the breast, exocrine pancreas, colon and prostate (Setyono-Han *et al.*, 1987; Schally, 1988; Parmar *et al.*, 1989; Weber *et al.*, 1989). Growth inhibition here could be mediated directly, via SSR which have been detected on breast, pancreas and prostate cancers (Reubi *et al.*, 1987; Setyono-Han *et al.*, 1987; Srkalovic *et al.*, 1990), or indirectly via suppression of local or circulating levels of growth factors (Schally, 1988).

There is some evidence that SS may play a role in growth regulation of small cell lung cancer (SCLC). SS-like immunoreactivity has been detected in SCLC cells (Sorensen *et al.*, 1981). SSR are expressed in SCLC tumours and cultured cells but not by non-SCLC (Taylor *et al.*, 1988*a*; Reubi *et al.*, 1990). The octapeptide SS analogue somatuline (BIM-23014C) has been shown to inhibit the growth of SCLC xenografts in immune-deprived mice (Taylor *et al.*, 1988*b*; Bogden *et al.*, 1990).

We have assessed the *in vitro* response of SCLC and non-SCLC cells to octreotide, correlating SSR expression with effects on growth and secretion of insulin-like growth factor-I (IGF-I), an autocrine growth factor for lung cancer cells (Minuto *et al.*, 1988; Macaulay *et al.*, 1988*a*; 1990). Results from these *in vitro* studies described below encouraged us to proceed to a clinical study, based on the fact that octreotide is a safe and well-tolerated drug (Parmar *et al.*, 1989; Reubi *et al.*, 1990). The main part of this study involved patients who had relapsed off-treatment after initial response to conventional chemotherapy. In addition we tried to assess octreotide in previously untreated patients, over a 7 day period during admission for staging investigations prior to conventional chemotherapy. In this latter group we hoped that serum neuron-specific enolase, a tumour marker for SCLC (Carney *et al.*, 1982), would be sufficiently sensitive to permit evaluation of response especially in the group treated for 7 days before chemotherapy. We also measured circulating levels of IGF-I. We have shown that serum IGF-I levels do not correlate with tumour bulk in patients with SCLC (Macaulay *et al.*, 1988*b*) and we used this parameter not as a tumour marker but rather as a measure of the endocrine effects of octreotide, as reported in acromegaly (Lamberts *et al.*, 1988).

### Patients and methods

### *Human lung cancer cells*

We obtained SCLC cell lines HC12 and HX149 from Dr G. Duchesne, Institute of Cancer Research, Surrey, NCI-H69 from Dr D. Carney, Mater Hospital, Dublin and non-SCLC cell lines NCI-H23 and -H226 from Dr A. Gazdar, National Cancer Institute, Bethesda, Maryland. SCLC cell line ICR-SC17 was established by us. Cell lines were characterised as previously described (Carney *et al.*, 1985; Duchesne *et al.*, 1987; Macaulay *et al.*, 1987) and were cultured in RPMI 1640 medium with 5% foetal calf serum (FCS). For establishment of xenografts, 2–5 × $10^6$ cells were innoculated subcutaneously into the flanks of female athymic (nu/nu) mice. Fresh tumour cells were obtained by fine needle aspiration of metastatic skin nodules in a patient with SCLC. The cells were taken into 1 ml unsupplemented RPMI medium, and a drop of the cell suspension was smeared on a slide and stained with Giemsa to confirm SCLC morphology. Half the remaining cell suspension was used in a $^{3}$Hthymidine incorporation assay (see below). Half was cultured in RPMI plus 5% FCS, and became established as cell line ICR-SC132.

Correspondence: I.E. Smith.
Received 7 January 1991; and in revised form 9 May 1991.

*Patients and treatment*

Twenty patients with histologically proven SCLC were entered into this study. Six patients were previously untreated, including three men and three women with a median age of 59.5 years (56–65 years). One patient had limited disease (LD, confined to one hemithorax and ipsilateral supraclavicular nodes) and five had extensive disease (ED). Fourteen patients were recruited on relapse following chemotherapy with ACE (Adriamycin, cyclophosphamide, etoposide) or MVC (methotrexate, vinblastine, carboplatin). They included 11 men and three women with a median age of 61.5 years (44–71). At presentation five had been staged as LD, and nine as ED. Before starting octreotide all patients were assessed by clinical examination, chest X-ray (CXR), full blood count (FBC) and biochemistry including liver function tests (LFTs). Serum was stored at −70°C for IGF-I and NSE assay. Other investigations (abdominal ultrasound scan, isotope bone scan, CT brain scan) were performed if clinically indicated.

Octreotide was administered subcutaneously: 50 μg 8-hourly for 24 h, 100 μg 8-hourly for 24 h, then 250 μg 8-hourly. In previously treated patients octreotide was continued to disease progression or development of unacceptable toxicity.

Gastrointestinal side-effect were treated with codeine phosphate 30–60 mg 4-hourly. If side-effects persisted, octreotide was given at a lower dose, and if necessary was discontinued. Staging investigations were repeated after 7 days in the group treated at presentation, and every 1–2 weeks in relapsed patients. At these intervals serum samples were obtained for NSE and IGF-I assays. Additional samples were obtained every 2 days from patients undergoing in-patient treatment before chemotherapy. Response was assessed by standard criteria (Miller *et al.*, 1981) and by serial serum levels of NSE and IGF-I. Side effects were graded as mild (grade 1), moderate (2), or severe (3).

*Growth assays*

Octreotide was provided by Dr P. Marbach, Sandoz, Basle. It was dissolved in sodium acetate buffer pH 6 with 0.1% bovine serum albumin (SMS buffer). $^3$H thymidine incorporation assays were performed in sterile 96-well microtitre plates (Macaulay *et al.*, 1988*a*). Single cell suspensions were preparing by syringing (SCLC) or trypsinisation (non-SCLC). Assays used cultured cells ($6 \times 10^3$/well) or fresh nucleated cells ($10^5$/well) obtained by fine needle aspirate of metastatic skin nodules. The cells were cultured in unsupplemented RMPI in the presence of octreotide $10^{-11}$–$10^{-6}$ M. Control wells received SMS buffer without octreotide. After 46 h incubation (37°C, 5% $CO_2$) the cells were labelled with $^3$H thymidine (Amersham) 0.4 μCi well, and after 24 h they were harvested and counted as previously described (Macaulay *et al.*, 1988*a*). For growth curves, cells were cultured in unsupplemented RPMI, RPMI plus TIS transferrin 100 μg ml$^{-1}$ or RPMI plus 5% FCS. Every 2–3 days, viable cell numbers were counted on a haemocytometer by trypan blue exclusion. After 2–4 days, when cell numbers were level or rising, cultures were treated with SMS buffer (controls) or octreotide $10^{-9}$ or $10^{-6}$ M. Samples of unconditioned and conditioned medium were stored at −20°C for later IGF-I assay. Growth assays on fresh tumour cells were performed once. Experiments on established cell lines were repeated at least twice, and the results of representative single experiments are given as the mean ± s.e.m. of triplicate estimations. Statistical assessment of differences between control and treatment groups used one-way analysis of variance and Dunnett's test (Zar, 1984).

*Somatostatin receptors (SSR)*

Cultures of human lung cancer cells in exponential growth phase were prepared by a method used to study epidermal growth factor receptor (Professor B. Gusterson, personal communication). Adherent monolayers (NCI-H23 and -H226) were detached by scraping into the medium, the cultures were centrifuged (2,000 r.p.m., 7 min) and the cell pellet was embedded in Tissue Tek OCT compound embedding medium (Ames, Indiana). Fresh tumour tissue was obtained by biopsy of subcutaneous nodules from one patient with SCLC before and at the end of octreotide treatment, and from mice bearing xenografts of HC12, HX149 and NCI-H226 on first passage *in vivo*. Cell pellets and tumours were snap frozen in liquid nitrogen and stored at −70°C. SSR were measured by autoradiography (ARG) on cryostat sections as previously described (Reubi *et al.*, 1990).

*Radioimmunoassays for NSE and IGF-I*

Serum NSE was measured on duplicate samples by radioimmunoassay (Pharmacia Ltd, Milton Keynes, UK). For IGF-I assay, unconditioned and conditioned media were centrifuged (1,000 r.p.m., 5 min), lyophilised, and reconstituted in distilled water at one tenth the original volume. Samples of medium and patients' serum underwent acid ethanol extraction with 2 N hydrochloric acid: absolute ethanol, 1:7 and were neutralised with Tris buffer. Duplicate aliquots were assayed using anti-IGF-I monoclonal antibody BPL-M23 as previously described (Teale & Marks, 1986; 1990; Macaulay *et al.*, 1990). Analysis of variance and Dunnett's test were used to assess the significance of differences in NSE and IGF-I levels before and during octreotide therapy.

### Results

*Established lung cancer cell lines*

$^3$H thymidine incorporation assays were performed on HX149 and HC12, to select octreotide doses for further study. Octreotide caused significant inhibition of $^3$H thymidine incorporation at $10^{-9}$ M in HX149 (63 ± 1% control, $P<0.05$; see Figure 1) and at $10^{-6}$ M in HC12 (87 ± 2%, $P<0.05$). We then tested the effect of octreotide $10^{-9}$ and $10^{-6}$ M on growth and IGF-I secretion by lung cancer cells in longer term culture. In HX149 growing in RPMI plus 5% FCS, cell numbers were lower in octreotide-treated cultures (Figure 2). Inhibition was greatest in the presence of octreotide $10^{-9}$ M, but the effects of both $10^{-9}$ and $10^{-6}$ M were significantly lower than control on days 9–16 ($P<0.01$). As we previously reported, HX149 secretes low levels of IGF-I, around 0.5 ng ml$^{-1}$. It was not possible to discern any octreotide-induced alteration in IGF-I secretion in the presence of 5% FCS, which provides around 2–5 ng ml$^{-1}$ IGF-I (Macaulay *et al.*, 1988*a*; 1990). We attempted to circumvent this problem by performing experiments in unsupplemented RPMI or RPMI plus TIS, but HX149 did not grow satisfactorily without serum. Octreotide $10^{-9}$ or $10^{-6}$ M had no effect on growth or IGF-I secretion by HC12, NCI-H69, ICR-SC17, NCI-H23 or NCI-H226.

**Figure 1** Effect of octreotide on $^3$H thymidine incorporation by SCLC cell line HX149.

**Figure 2** Effect of octreotide $10^{-9}$ M (▲) or $10^{-6}$ M (■) on SCLC cell line HX149 in RPMI plus 5% FCS.

Receptor autoradiography of xenograft tissue showed weakly positive SSR expression in HX149 and no detectable SSR in HC12 or NCI-H226. SSR were also detectable in cell pellets of NCI-H69, HX149 and ICR-SC17, but not in HC12, NCI-H23 or -H226. In NCI-H69 SSR were homogenously distributed at high density, whereas SSR expression by HX149 and ICR-SC17 was weak and patchy.

*Clinical study in patients with SCLC*

The six previously untreated patients with SCLC were treated with octreotide for a median 7 days (range 1–11 days). The 14 patients with slow-tempo relapse after chemotherapy received median 17 days treatment (2–104 days). Octreotide was administered on an out-patient basis by the patients themselves (seven cases), a relative (three), or district nurses (one, treated with 375 µg bd). Four in-patients received injections from ward nurses.

Five of six previously untreated patients had no change in tumour bulk during the week of therapy. One had progressive disease (PD) in mediastinal lymph nodes, with the development of stridor. Two of 14 relapsed patients had stable disease (SD) accompanied by relief of respiratory symptoms, and were able to continue octreotide for 8 and 15 weeks. Of 12 patients treated for less then 1 month, one stopped treatment after 2 days and was not evaluable for response, and the others had progressive disease (see Table I). One patient with multiple metastatic skin nodules consented to further studies on the effect of octreotide on tumour cell growth and SSR expression (see below).

Serum was obtained for NSE and IGF-I assay in all patients before starting octreotide and serial samples were available in 18. Five of six previously untreated patients had high baseline NSE levels ($>12$ ng $ml^{-1}$) and of four with serial measurements one remained high, two fell slightly (39.5 to 29.5 and 14 to 10 ng $ml^{-1}$; $P<0.05$ in each case) and one rose significantly from 72 (pre-octreotide) to 200 (on treatment; $P<0.05$) to 78 (end of treatment). An early 'surge' in NSE has been previously noted in response to chemotherapy, where it may represent rapid destruction of NSE-producing cells (Nou *et al.*, 1990). Thirteen of 14 relapsed patients had high pretreatment serum NSE levels. Of 12 with serial measurements, NSE levels fell from 28 to 9 ng $ml^{-1}$ in one case ($P<0.01$). In six patients there was no statistically significant alteration in NSE levels although all had PD clinically. Five patients had a significant rise ($P<0.01$) in serum NSE while on octreotide, including four with clinical PD and one who had stable disease until the end of 15 weeks octreotide.

Pre-octreotide serum IGF-I levels were normal in 17/20 patients. Low levels ($<0.2$ u $ml^{-1}$) in three cases were probably related to abnormal liver function (D'Ercole *et al.*, 1984). Serum IGF-I was suppressed during treatment with octreotide in 15 of 18 patients who had serial measurements. Serum IGF-I levels fell in all five previously-untreated patients where serial samples were obtained ($P<0.05$ in two cases, $P<0.01$ in three). This includes one patient in whom IGF-I levels on octreotide were, at 0.07–0.13 u $ml^{-1}$, below the normal range ($>0.2$ u $ml^{-1}$). Of 13 relapsed patients with serial measurements, IGF levels were unchanged in four and fell in eight ($P<0.05$ in two cases, $P<0.01$ in six). One patient had a significant rise in serum IGF-I (0.22–0.56 u $ml^{-1}$, $P<0.01$) possibly related to an improvement in nutritional status accompanied by symptomatic benefit and stable disease clinically. Overall the change in IGF-I, expressed as the lowest level on octreotide as a per cent of the pre-treatment value, was median 53%, mean $62 \pm 7\%$ (range 23–150%).

Octreotide was well tolerated by most patients. Details of symptomatic toxicity are given in Table II. Ten patients experienced local pain at the site of administration. In one this led to cessation of therapy, but in the rest it was ameliorated by giving the injection slowly. Eleven patients had gastrointestinal side-effects which were generally mild, but which necessitated dose reduction to 100 µg tds in two cases and cessation of therapy in one case. One patient accidentally administered 1,000 µg instead of 100 µg octreotide on the second day of therapy. She experienced vomiting (grade 2) and 12 h of severe (grade 3) abdominal cramps and diarrhoea. These symptoms settled within 12–24 h, and she was able to continue treatment at 250 µg tds for 8 weeks. These were no treatment-related changes in FBC or LFTs.

Four of six previously untreated patients completed the planned 7 days of octreotide therapy. Treatment was discontinued early in two cases, one for toxicity and the other for PD. All six patients went on to receive combination chemotherapy in a randomised trial comparing ACE with MVC. Five of the six responded to first-line chemotherapy, and all responded to first or second-line chemotherapy. This group had a median survival of 36 weeks (range 20–51) from the start of treatment. Octreotide was discontinued in one relapsed patients because of toxicity (local pain), and in the remaining 13 because of PD. From the start of octreotide therapy this group had a median survival of only 13 weeks (3–50).

*Experimental studies in one patient with relapsed SCLC*

A patient with SCLC (LD), inappropriate antidiuretic hormone secretion, Lambert-Eaton myasthenic syndrome and autonomic neuropathy who had relapsed after ACE chemotherapy with metastatic skin/subcutaneous nodules, consented to nodule biopsy and aspiration before and 2 weeks after starting octreotide. During this period his tumour progressed. Changes in serum NSE and IGF-I are shown in Figure 3.

**Table I** Clinical response to octreotide in 20 patients with SCLC

| | *CR/PR* | *SD* | *PD* | *NE* | *Total* |
|---|---|---|---|---|---|
| Previously untreated | 0 | 0 | 1 | 5 | 6 |
| Relapsed after chemotherapy | 0 | 2 | 11 | 1 | 14 |

CR – complete response; PR – partial response; SD – stable disease; PD – progressive disease; NE – not evaluable.

**Table II** Side-effects of octreotide

| | *Grade* | | | |
|---|---|---|---|---|
| *Symptom* | *0* | *1* | *2* | *3* |
| Local pain | 10 | 7 | 1 | 2 |
| Local erythema | 17 | 3 | 0 | 0 |
| Anorexia | 18 | 1 | 1 | 0 |
| Nausea/vomiting | 18 | 0 | 2 | 0 |
| Abdominal bloating/cramps | 13 | 4 | 2 | 1 |
| Diarrhoea | 10 | 5 | 4 | 1 |

The table gives numbers of patients experiencing each symptom; worst grade at any time during treatment.

**Figure 3** Effect of treatment with octreotide 250 μg tds on serum levels of NSE (●) and IGF-I (▲) in patient with relapsed SCLC. Normal ranges: NSE < 12 ng ml$^{-1}$, IGF-I 0.2–1.2 u ml$^{-1}$.

The pre-octreotide skin nodule biopsy showed metastatic SCLC which expressed detectable SSR by ARG. Fine needle aspiration of another skin lesion yielded fresh nucleated cells of which 85% had typical SCLC morphology and the rest were mature lymphocytes. Half the cells were incubated in unsupplemented RPMI with octreotide $10^{-11}$–$10^{-6}$ M which had no effect on $^3$H thymidine incorporation. The remaining cells were cultured in RPMI plus 5% FCS and grew as floating clusters, becoming established as SCLC cell line ICR-SC132. After approximately 15 passages *in vitro* cultures of ICR-SC132 in RPMI plus 5% FCS were grown in the presence and absence of octreotide $10^{-9}$ or $10^{-6}$ M. There was no evidence of growth inhibition or suppression of IGF-I secretion. The post-octreotide skin nodule biopsy retained typical features of SCLC and detectable SSR by ARG (Figure 4). Fresh tumour cells obtained by fine needle aspiration again showed no inhibition of $^3$H thymidine incorporation in the presence of octreotide $10^{-11}$–$10^{-6}$ M. This sample did not establish as a cell line.

### Discussion

In this study SSR were expressed by 3/4 established SCLC cell lines but only 1/4 showed growth inhibition in response to octreotide. This effect occurred in HX149 which expresses SSR, albeit weakly. We observed maximum inhibition in the presence of octreotide $10^{-9}$ M, the concentration which is achieved clinically following a single subcutaneous injection of octreotide (del Pozo *et al.*, 1986). The dose-response relationship in $^3$H-thymidine incorporation assay was similar to the 'bell-shaped' curve seen in cultured breast cancer cells (Setyono-Han *et al.*, 1987). This could have important implications for the clinical assessment of SS analogues, since the maximum tolerated dose may not necessarily have the greatest antitumour effect. None of our SSR negative cell lines were inhibited by octreotide, but neither was NCI-H69 which expresses high levels of SSR. However growth of this cell line was inhibited *in vitro* by two other octapeptide SS analogues, somatuline (BIM-23014C) and RC-160 (Taylor *et al.*, 1988*b*, Dr A. Schally, personal communication). Somatuline also inhibited the growth of NCI-H69 xenografts *in vivo*, especially when adminstered by perilesional infusion (Bogden *et al.*, 1990).

This clinical study was prompted by our *in vitro* data together with reports from other groups demonstrating experimental and clinical activity of SS analogues in NE tumours including SCLC. In using a brief course of octreotide in previously-untreated patients, we aimed to assess its effects first-line without delaying conventional treatment. None of six patients responded to octreotide but all subsequently responded to combination chemotherapy. This is important since it has been suggested that front-line treatment with new agents may impair response to conventional therapy given second-line (Cullen *et al.*, 1987). We extended the study to patients with slow-tempo relapse after chemotherapy, to assess more prolonged administration in terms of effects on tumour bulk and feasibility as out-patient treatment. Octreotide was generally well tolerated, but most patients had PD within a few weeks and only two patients were able to continue treatment for more than one month. There was no evidence of clinical antitumour activity in any patient, including one whose tumour was shown to express SSR before and after treatment.

**Figure 4** Skin nodule biopsy in patients with relapsed SCLC after 2 weeks treatment with octreotide. **a**, H&E, **b**, Total binding of $^{125}$I Tyr-3-octreotide, **c**, Non-specific binding.

We hoped that serial measurements of serum NSE would be sufficiently sensitive to detect small changes in tumour bulk. However, short-term changes in NSE correlated poorly with clinical status. Since this study began, others have questioned the value of serum NSE in monitoring response to treatment, because in individual patients it adds little to standard clinical staging (Carney & Teeling, 1988; Nou *et al.*, 1990).

Serum IGF-I levels fell in most patients, probably reflecting suppression by octreotide of physiological (mainly hepatic) IGF-I secretion, as occurs in diabetics (Plewe *et al.*, 1987). In acromegalics treated with octreotide, serum IGF-I correlates well with levels of growth hormone and achievement of biochemical 'cure' (Lamberts *et al.*, 1988). This suggests that we had used an effective dose of octreotide, at least in endocrine terms. A recently reported study of eight patients with solid tumours (including cancers of the breast, pancreas, colon, kidney and ovary) showed similar reduction in circulating IGF-I levels on octreotide, although there were no data on clinical response, and suggested that suppression of serum IGF-I is in itself a promising approach to treatment (Pollack *et al.*, 1989). However it seems unlikely that we could suppress secretion of IGF-I or other growth factors, especially those synthesised by the tumour cells themselves, to the point where their local tissue concentration would limit neoplastic growth. Any growth inhibition seems more likely to be mediated by a direct effect of SS analogues on the tumour cells themselves.

In summary, although octreotide has documented antitumour activity in other SSR positive NE tumours, we saw little correlation between SSR expression and growth inhibition in SCLC, either *in vitro* or clinically. This may be because the SSR detected on SCLC membranes do not mediate a growth inhibitory response, as has been demonstrated in meningiomas (Reubi *et al.*, 1989). Alternatively we may have used a suboptimal dose or schedule of octreotide. If the dose-reponse curve is genuinely 'bell-shaped', as has been suggested in breast cancer (Setyono-Han *et al.*, 1987), the maximum tolerated dose may not necessarily have the greatest antiproliferative activity. Therefore it may be worth assessing other doses/schedules of octreotide administration, and other SS analogues reported to have pre-clinical activity in SCLC. In particular, analogues of the RC series including RC-160 have been shown to bind with higher affinity than octreotide to SSR on human tumour membranes (Srkalovic *et al.*, 1990). The RC analogues, but not octreotide have also been shown to stimulate dephosphorylation if the EGF receptor in pancreatic cancer cells (Liebow *et al.*, 1989). This mechanism is not likely to be relevant in SCLC cells, which lack EGF receptors (Reubi *et al.*, 1990). Nevertheless, these studies suggest that some SS analgoues might be superior to others in terms of direct, receptor-mediated antitumour activity.

This work was supported by the Alan Jay Lerner fund for lung cancer research, and by the T.K. Stubbins Fellowship of the Royal College of Physicians. We thank B. Waser for excellent technical assistance, and Mrs Julia Holborn for preparing the manuscript.

### References

BLOOM, S.R. & POLAK, J.M. (1987). Somatostatin. *Br. Med. J.*, **295**, 288.

BOGDEN, A.E., TAYLOR, J.E., MOREAU, J.-P., COY, D.H. & LE PAGE, D.J. (1990). Response of human lung tumor xenografts to treatment with a somatostatin analogue (somatuline). *Cancer Res.*, **50**, 4360.

CARNEY, D.N., IHDE, D.C., COHEN, M.H. & 4 others (1982). Serum neuron-specific enolase: a marker for disease extent and response to therapy of small-cell lung cancer. *Lancet*, **i**, 583.

CARNEY, D.N., GAZDAR, A.F., BEPLER, G. & 5 others (1985). Establishment and characterisation of small cell lung cancer cell lines having classic and variant features. *Cancer Res.*, **45**, 2913.

CARNEY, D.N. & TEELING, M. (1988). Neuron-specific enolase: how useful as a cancer marker? *Eur. J. Cancer Clin. Oncol.*, **24**, 825.

COMI, R.J. (1989). Pharmacology and use in pituitary tumors, pp 36–41. In Gorden, P. moderator. Somatostatin and somatostatin analogue (SMS 201-995) in treatment of hormone secreting tumors of the pituitary and gastrointestinal tract and non-neoplastic diseases of the gut. *Ann. Intern. Med.*, **110**, 35.

CULLEN, M.H., SMITH, S.R., BENFIELD, G.F. & WOODROFFE, C.M. (1987). Testing new drugs in untreated small cell lung cancer may prejudice the results of standard treatment: a phase II study of oral idarubicin in extensive disease. *Cancer Treat. Rep.*, **71**, 1277.

DEL POZO, E., NEUFELD, M., SCHLUTER, K. & 8 others (1986). Endocrine profile of a long-acting somatostatin derivative SMS 201-995. Study in normal volunteers following subcutaneous administration. *Acta Endocrinol.*, **111**, 433.

D'ERCOLE, A.J., STILES, A.D. & UNDERWOOD, L.E. (1984). Tissue concentrations of somatomedin C: further evidence for multiple sites of synthesis and paracrine or autocrine mechanisms of action. *Proc. Natl Acad. Sci. USA*, **81**, 935.

DUCHESNE, G.M., EADY, J.J., PEACOCK, J.H. & PERA, M.F. (1987). A panel of human lung carcinoma lines: establishment, properties and common characteristics. *Br. J. Cancer*, **56**, 287.

FROHMAN, L.A. & KRIEGER, D.T. (1987). Neuroendocrine physiology and disease. In *Endocrinology and Metabolism*, Felig, P., Baxter, J.D., Broadus, A.E. & Frohman, L.A. pp. 185–247, New York, 2nd Ed.

KRAENZLIN, M.E., CHANGE, J.C., WOOD, S.M., CARR, D.H. & BLOOM, S.R. (1985). Long term treatment of a VIPoma with somatostatin analogue resulting in remission of symptoms and possible shrinkage of metastases. *Gastroenterology*, **88**, 185.

KVOLS, L.K., MOERTEL, C.G., O'CONNELL, M.J., SCHUTT, A.J., RUBIN, J. & HAHN, R.G. (1986). Treatment of the malignant carcinoid syndrome. Evaluation of a long-acting somatostatin analogue. *N. Engl. J. Med.*, **315**, 663.

LAMBERTS, S.W.J. & UITTERLINDEN, P. & DEL POZO, E. (1987). SMS 201-995 induces a continuous decline in circulating growth hormone and somatomedin-C levels during therapy of acromegalic patients for over two years. *J. Clin. Endocrinol. Metabol.*, **65**, 703.

LAMBERTS, S.W.J., UITTERLINDEN, P., SCHUJFF, P.C. & KLIJN, J.G.M. (1988). Therapy of acromegaly with Sandostatin: the predictive value of an acute test, the value of serum somatomedin-C measurements in dose adjustment and the definition of a biochemical 'cure'. *Clin. Endocrinol.*, **29**, 411.

LIEBOW, C., REILLY, C., SERRANO, M. & SCHALLY, A.V. (1989). Somatostatin analogues inhibit growth of pancreatic cancer by stimulating tyrosine phosphatase. *Proc. Natl Acad. Sci. USA*, **86**, 2003.

MACAULAY, V., JOSHI, G.P., EVERARD, M., SMITH, I.E. & MILLAR, J.L. (1987). A high molecular weight non-bombesin/gastrin releasing peptide growth factor in small cell lung cancer. *Br. J. Cancer*, **56**, 791.

MACAULAY, V.M. & LOVER, G.H. (1990). New angles on ligand: receptor interaction. *Cancer Mol. Biol.*, **3**, 21.

MACAULAY, V.M., TEALE, J.D., EVERARD, M.J., JOSHI, G.P., SMITH, I.E. & MILLAR, J.L. (1988*a*). Somatomedin-C/insulin-like growth factor-I is a mitogen for human small cell lung cancer. *Br. J. Cancer*, **57**, 91.

MACAULAY, V.M., TEALE, J.D., EVERARD, M.J., JOSHI, G.P., MILLAR, J.L. & SMITH, I.E. (1988*b*). Serum insulin-like growth factor-I levels in patients with small cell lung cancer. *Eur. J. Cancer Clin. Oncol.*, **24**, 1241.

MACAULAY, V.M., EVERARD, M.J., TEALE, J.D. & 4 others (1990). Autocrine function for insulin-like growth factor 1 in human small cell lung cancer cell lines and fresh tumor cells. *Cancer Res.*, **50**, 2511.

MATON, P.N. (1989). Use in patients with gut neuroendocrine tumours, pp. 41–44. In Gorden, P., moderator. Somatostatin and somatostatin analogue (SMS 201-995) in treatment of hormone-secreting tumors of the pituitary and gastrointestinal tract and non-neoplastic diseases of the gut. *Ann. Intern. Med.*, **110**, 35.

MILLER, A.B., HOOGSTRATEN, B., STAQUET, M., WINKLER, A. (1981). Reporting results of cancer treatment. *Cancer*, **47**, 207.

MINUTO, F., DEL MONTE, P., BARRECA, A., ALAMA, A., CARIOLA, G. & GIORDANO, G. (1988). Evidence for autocrine mitogenic stimulation by somatomedin-C/ insulin-like growth factor I on an established human lung cancer cell line. *Cancer Res.*, **48**, 3716.

MOREAU, J.P. & DEFEUDIS, F.V. (1987). Pharmacological studies of somatostatin and somatostatin-analogues: therapeutic advances and perspectives. *Life Sci.*, **40**, 419.

NOU, E., STEINHOLTZ, L., BERGH, J., NILSSON, K. & PAHLMAN, S. (1990). Neuron-specific enolase as a follow-up marker in small cell bronchial carcinoma. A prospective study in an unselected series. *Cancer*, **65**, 1380.

PARMAR, H., BOGDEN, A., MOLLARD, M., DE ROUGE, B., PHILIPS, R.H. & LIGHTMAN, S.L. (1989). Somatostatin and somatostatin analogues in oncology. *Cancer Treat. Rev.*, **16**, 95.

PLEWE, G., NOELKEN, G., KRAUSE, U. & 2 others (1987). Suppression of growth hormone and somatomedin C by long-acting somatostatin analog SMS 201-995 in Type I diabetes mellitus. *Hormone Res.*, **27**, 7.

POLLACK, M.N., POLYCHRONAKOS, C. & GUYDA, H. (1989). Somatostatin analogues SMS 201-995 reduces serum IGF-I levels in patients with neoplasms potentially dependent on IGF-I. *Anticancer Res.*, **9**, 889.

REUBI, J.C., RIVIER, J., PERRIN, M., BROWN, M. & VALE, W. (1982). Specific high affinity binding sites for somatostatin-28 on pancreatic β-cells: differences with brain somatostatin receptors. *Endocrinol.*, **110**, 1049.

REUBI, J.C. & LANDOLT, A.M. (1984). High density of somatostatin receptors in pituitary tumors from acromegalic patients. *J. Clin. Endocrinol. Metab.*, **59**, 1148.

REUBI, J.C., MAURER, R., VON WERDER, J., TORHORST, J., KLIJN, J.G. & LAMBERTS, S.W. (1987). Somatostatin receptors in human endocrine tumors. *Cancer Res.*, **47**, 551.

REUBI, J.C., HORISBERGER, U., LANG, W., KOPER, J.W., BRAAKMAN, R. & LAMBERTS, S.W.J. (1989). Coincidence of EGF receptors and somatostatin receptors in meningiomas but inverse, differentiation-dependent relationship in glial tumors. *Am. J. Pathol.*, **134**, 337.

REUBI, J.C., WASER, B., SHEPPARD, M. & MACAULAY, V.M. (1990). Somatostatin receptors are present in small cell but not in non-small cell primary lung carcinomas: relationship to EGF-receptors. *Int. J. Cancer*, **45**, 269.

SCHALLY, A.V. (1988). Oncological applications of somatostatin analogues. *Cancer Res.*, **48**, 6977.

SETYONO-HAN, B., HENKELMAN, M.S., FOEKENS, J.A. & KLIJN, J.G.M. (1987). Direct inhibitory effects of somatostatin (analogues) on the growth of human breast cancer cells. *Cancer*, **47**, 156.

SORENSON, G.D., PETTENGILL, O.S., BRINCK-JOHNSEN, T., CATE, C.C. & MAURER, L.H. (1981). Hormone production by cultures of small-cell carcinoma of the lung. *Cancer*, **47**, 1289.

SRKALOVIC, G., REN-ZHI, C. & SCHALLY, A.V. (1990). Evaluation of receptors for somatostatin in various tumours using different analogs. *J. Clin. Endocrinol. Metabol.*, **70**, 661.

TAYLOR, J.E., COY, D.H. & MOREAU, J.-P. (1988*a*). High affinity binding of [$^{125}$I-Try$^{11}$]somatostatin-14 to human small cell lung carcinoma (NCI-H69). *Life Sci.*, **43**, 421.

TAYLOR, J.E., BOGDEN, A.E., MOREAU, J.-P. & COY, D.H. (1988*b*). *In vitro* and *in vivo* inhibition of human small cell lung carcinoma (NCI-H69) growth by a somatostatin analogue. *Biochem. Biophys. Res. Commun.*, **153**, 81.

TEALE, J.D. & MARKS, V. (1986). The measurement of insulin-like growth factor I: clinical applications and significance. *Ann. Clin. Biochem.*, **23**, 413.

TEALE, J.D. & MARKS, V. (1990). Inappropriately elevated plasma insulin-like growth factor II in relation to suppressed insulin-like growth factor I in the diagnosis of non-islet cell tumour hypoglycaemia. *Clin. Endocrinol.*, **33**, 87.

WEBER, C., MERRIAM, L., KOSCHITZKY, T. & 4 others (1989). Inhibition of growth of human breast carcinoma *in vivo* by somatostatin analog SMS 201-995: treatment of nude mouse xenografts. *Surgery*, **106**, 416.

WOOD, S.M., KRAENZLIN, M.E., ADRIAN, T.E. & BLOOM, S.R. (1985). Treatment of patients with pancreatic endocrine tumors using a new long-acting somatostatin analogue: syptomatic and peptide responses. *Gut*, **26**, 436.

ZAR, J.H. (1984). *Biostatistical Analysis*, 2nd Ed, Prentice-Hall: Englewood Cliffs, NJ.